# Pulsed-Focused Ultrasound Slows B16 Melanoma and 4T1 Breast Tumor Growth through Differential Tumor Microenvironmental Changes

**DOI:** 10.3390/cancers13071546

**Published:** 2021-03-27

**Authors:** Gadi Cohen, Parwathy Chandran, Rebecca M. Lorsung, Omer Aydin, Lauren E. Tomlinson, Robert B. Rosenblatt, Scott R. Burks, Joseph A. Frank

**Affiliations:** 1 Frank Laboratory, Radiology and Imaging Sciences, Clinical Center, National Institutes of Health, 10 Center Drive, Bethesda, MD 20892-1074, USA; Rebecca.Lorsung@som.umaryland.edu (R.M.L.); biomer@umich.edu (O.A.); lauren.tomlinson@nih.gov (L.E.T.); robert.rosenblatt@nih.gov (R.B.R.); scott.burks@nih.gov (S.R.B.); 2 Department of Biomedical Engineering, Erciyes University, Kayseri 38039, Turkey; 3 National Institute of Biomedical Imaging and Bioengineering, National Institutes of Health, 10 Center Drive, Bethesda, MD 20892-1074, USA

**Keywords:** focused ultrasound, B16 melanoma, 4T1 breast cancer, immune response, tumor microenvironment

## Abstract

**Simple Summary:**

This study provides a conceptual overview correlating temporal changes in the tumor immune-microenvironment (TIME) to the non-ablative pulse focused ultrasound (pFUS) in two different types of tumors. The pFUS-induced immunomodulation revealed differential anti-tumor molecular and cellular responses between the two tumor types, demonstrating tumor-type-dependent responses to identical pFUS sonication parameters. It also supports the potential use of pFUS as a possible adjuvant to ablative tumor treatment to elicit enhanced anti-tumor responses and slow tumor growth.

**Abstract:**

Focused ultrasound (FUS) has shown promise as a non-invasive treatment modality for solid malignancies. FUS targeting to tumors has been shown to initiate pro-inflammatory immune responses within the tumor microenvironment. Pulsed FUS (pFUS) can alter the expression of cytokines, chemokines, trophic factors, cell adhesion molecules, and immune cell phenotypes within tissues. Here, we investigated the molecular and immune cell effects of pFUS on murine B16 melanoma and 4T1 breast cancer flank tumors. Temporal changes following sonication were evaluated by proteomics, RNA-seq, flow-cytometry, and histological analyses. Proteomic profiling revealed molecular changes occurring over 24 h post-pFUS that were consistent with a shift toward inflamed tumor microenvironment. Over 5 days post-pFUS, tumor growth rates were significantly decreased while flow cytometric analysis revealed differences in the temporal migration of immune cells. Transcriptomic analyses following sonication identified differences in gene expression patterns between the two tumor types. Histological analyses further demonstrated reduction of proliferation marker, Ki-67 in 4T1, but not in B16 tumors, and activated cleaved-caspase 3 for apoptosis remained elevated up to 3 days post-pFUS in both tumor types. This study revealed diverse biological mechanisms following pFUS treatment and supports its use as a possible adjuvant to ablative tumor treatment to elicit enhanced anti-tumor responses and slow tumor growth.

## 1. Introduction

Solid tumors are a dynamic compilation of malignant cells, stem cells along with infiltrating immune cells, vasculature, stroma, extracellular matrix, and soluble factors that together constitute the tumor microenvironment [1,2,3]. The tumor immune microenvironment (TIME) reflects interactions between tumor cells and the immune system, which are mediated by damage-associated molecular patterns (DAMP); cytokines, chemokines, and trophic factors (CCTF); and cell adhesion molecules (CAM) [1]. Together, they regulate tumor growth, metastatic dissemination, and therapeutic outcomes [4,5].

TIME can be described immunologically as infiltrated-inflamed (I-I) ‘hot’ or infiltrated-excluded (I-E) ‘cold’ microenvironment [1,2]. Hot TIME is primarily pro-inflammatory and anti-tumor, containing cytotoxic T cells (CTL), helper T cells (T_h1_), dendritic cells (DC), natural killer cells (NK), B cells, and M_1_ macrophages [6,7]. Hot TIME also express tumor necrosis factor alpha (TNFα), interferon gamma (IFNγ), IFNγ-induced protein-10 (IP-10/CXCL10), interleukin (IL) 1α, IL1β, IL2, and IL17, and endothelial CAM [8]. Alternatively, cold TIME are generally immunosuppressive and promote tumor growth. They contain regulatory T cells (T_reg_), myeloid-derived suppressor cells (MDSC), tumor-associated macrophages [9,10], and cancer-associated fibroblasts [11]. Cold TIME express transforming growth factor beta (TGFβ), IL4, IL10, IL13, and macrophage inflammatory protein 1a (MIP1a/CCL3), monocyte chemoattractant protein 1 (MCP1/CCL2), vascular endothelial growth factor (VEGF) [12,13]. Solid tumors are frequently heterogeneous with variable regions of ‘hot’ and ‘cold’ TIME and the primary goal of immune-based tumor therapies is to shift the balance toward a ‘hot’ TIME by inducing anti-tumor immune responses [14,15].

Thermal ablation by image-guided high intensity focused ultrasound (HIFU) is an FDA-approved treatment procedure for benign or malignant cancers [16] that in combination with immunomodulatory cells or biological therapeutics can result in an anti-tumor TIME [17,18,19,20]. Alternatively, non-ablative pulsed focused ultrasound (pFUS) induces numerous molecular and cellular responses in sonicated tissues [21] and has potential as an immunomodulatory adjuvant in tumor therapy. The mechanotransducive effect of pFUS occurs through acoustic radiation and cavitation forces that alter expression of CCTF and CAM within tissues [21,22,23,24]. We previously demonstrated pFUS to modulate immune cell phenotypes, CCTF, and CAM expression in B16 and 4T1 flank tumors at 24 h [25,26]. In both tumor types, TIME shifted from I-E towards I-I following pFUS and was associated with slowing of tumor growth. However, the mechanisms behind the altered tumor growth and TIME by pFUS remains poorly understood.

In this study, we investigated the temporal changes following non-ablative pFUS on CCTF, CAM, immune cell profiles, and transcriptomics of murine B16 melanoma and 4T1 breast cancer flank tumors, along with immune cell changes in spleens (Sp) and inguinal lymph nodes (LN) up to 5 days post-sonication compared to un-sonicated time-matched controls (TMC). The temporal dynamics of TIME following pFUS revealed differential anti-tumor molecular and cellular responses between the two tumor types, demonstrating tumor-type-dependent responses to identical pFUS sonication parameters.

## 2. Materials and Methods

### 2.1. Cell Culture

B16 melanoma cells (B16-F0: ATCC^®^ CRL-6322^TM^; RRID: CVCL_0604) and 4T1 breast cancer cells (ATCC^®^ CRL-2539; RRID: CVCL_0125) were cultured in Dulbecco’s modified Eagle medium (DMEM) for B16 cells or Roswell Park Memorial Institute (RPMI) 1640 medium for 4T1 cells (Gibco, Paisley, UK). Both media contained 10% fetal bovine serum (FBS) and 1% penicillin-streptomycin (Gibco). Cells were maintained at 37 °C under 5% CO_2_ and were passaged (up to 10 passages) at 75–90% confluency using TyrpLE Express Enzyme (Gibco).

### 2.2. Murine B16 and 4T1 Tumor Models

**Figure 1:**
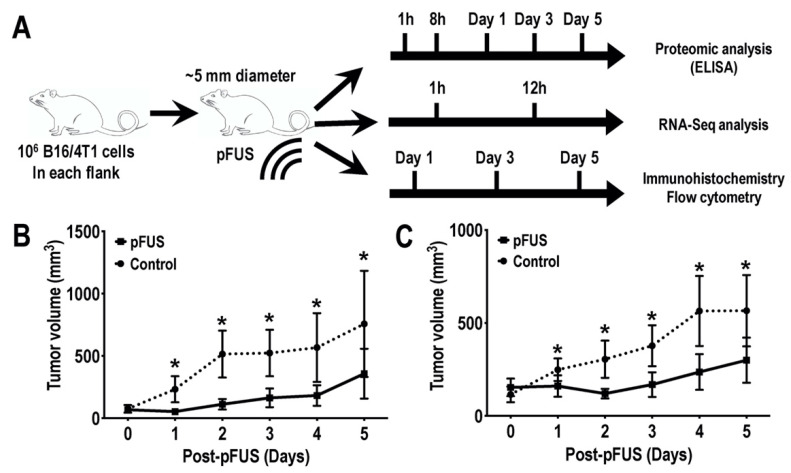
(**A**) Schematic diagram of the experimental set-ups. Murine melanoma B16 or breast 4T1 cells were inoculated into C57BL/6 or BALB/c mice (*n* = 6 mice/tumor type/time point), respectively. Mice were treated with 6 MPa pFUS once tumor sizes reached ~5 mm in diameter. Tumors were harvested at different time points post-pFUS and were subjected to proteomic (top), transcriptomic (middle), immunohistochemistry, and flow cytometry (bottom) analyses. Tumor size volumes of (**B**) B16 melanoma (**C**) or 4T1 breast flank tumor model acquired from the third cohort study. Black lines indicate tumor size (mean ± SD) following pFUS treatment on day 0. Dashed black lines indicated an untreated control group. Asterisks indicate statistical significance (*p* < 0.05; *t*-test).

Animal procedures were conducted according to the National Research Council’s Guide for the Care and Use of Laboratory [27] and approved by the Animal Care and Use Committee of the National Institutions of Health Clinical Center (LDRR-1703). Female C57BL/6 (*n* = 72; IMSR cat. no. CRL:027) and BALB/c (*n* = 72; IMSR cat. no. CRL:028) mice (8–12 weeks old; Charles River Laboratories) had free access to food and water. Depilatory cream was used to remove hair 24 h before tumor inoculation. Mice received bilateral subcutaneous implantations of mycoplasma-free 10^6^ B16 cells (in C57BL/6) or 4T1 cells (in BALB/c mice) suspended in 100 μL phosphate buffered saline (PBS). During procedures, mice were anesthetized with 1.5–3% isoflurane. Tumor volumes were measured by digital caliper and reached an average diameter of 5 mm before pFUS treatment (Figure 1A).

### 2.3. pFUS Treatment

Ultrasound-guided pFUS treatments were performed as previously described [25,26]. Briefly, non-ablative pFUS (VIFU 2000; Alpinion, Seoul, Korea) was administered in degassed water at 37 °C with a single-element focused transducer (1.15 MHz; 1.2 mm focal diameter and 6 dB cutoff) to tumors of randomly selected mice. pFUS used the following parameters: 6 MPa peak negative pressure (I_SATP_ = 2683 W/cm^2^; mechanical index = 5.6) 10% duty cycle, 5 Hz pulse repetition frequency, 100 pulses per site. Entire tumor volumes were sonicated with elemental spacing of 2 mm. Control tumors from separate sets of animals received sham sonication (transducer power = 0 W).

### 2.4. Proteomic Analyses

Tumors (*n* = 6 mice/tumor type/time point) were flash-frozen in liquid N_2_ and stored at −80 °C until use. Tumors were next homogenized in lysis buffer containing 0.05% Tween-20 and protease inhibitors (Millipore-Sigma, Burlington, MA, USA). Protein concentrations were determined by bicinchoninic acid assay (ThermoFisher Scientific, Waltham, MA, USA). Proteomic analysis was performed using a Mouse Cytokine Chemokine 32-plex Magnetic Bead Panel (Millipore-Sigma, MCYTMAG-70-PX32) on a Bio-Plex 200 System (Bio-Rad, Hercules, CA, USA). Additional single-plex ELISA (R&D Systems) for intracellular adhesion molecule-1 (ICAM-1/CD54; DY796), IFNγ (DuoSet; DY485), vascular cell adhesion molecule-1 (VCAM-1/CD106; DY643), and TGF-β1 (MB100B/SMB100B) were performed according to manufacturer’s protocols.

### 2.5. Flow Cytometric Analyses

Tumors were harvested (*n* = 6 mice/tumor type/time point) and dissociated into single-cell suspensions by enzymatic digestion in Hanks’ balanced salt solution (HBSS) (Gibco) containing 1% collagenase (Sigma-Aldrich, St. Louis, MO, USA), 2% FBS, and 1% penicillin-streptomycin for 60 min at 37 °C. Suspensions were mechanically agitated and passed through 70-μm strainers (BD Biosciences, San Jose, CA, USA). Spleens (Sp), and inguinal LN were suspended in an ammonium-chloride-potassium lysing buffer (ThermoFisher Scientific) and passed through 70-μm cell strainers. Cells were washed in PBS then fixed for 15 min using 4% paraformaldehyde. For immunostaining, 10^6^ cells from Sp, LN, or tumor samples were treated with an Fc receptor-blocking antibody (BioLegend, San Diego, CA, USA, anti-mouse CD16/32) in staining buffer (PBS containing 0.5% bovine serum albumin (BSA) and 2 mM ethylenediaminetetraacetic acid (EDTA)) for 10 min, followed by primary antibodies (Table S1) for 45 min on ice. Ten thousand events were acquired from each sample on an Accuri^TM^ C6 Cytometer (BD Biosciences) and data was processed using FlowJo software.

### 2.6. Immunohistochemistry Analysis (IHC)

Tumors were harvested and fixed in 4% paraformaldehyde in PBS for 24 h at 4 °C. Tumors were embedded in paraffin and sectioned to 6-micron thickness. Following incubation at 65 °C for 1 h, sections (*n* = 4–6 sections/tumor type/time point) were deparaffined and rehydrated. Antigen retrieval was performed in citric acid (pH = 6.0) buffer (Vector Laboratories, Burlingame, CA, USA) at 100 °C for 20 min. Sections were then blocked in SuperBlock^TM^ (ThermoFisher Scientific) for 20 min and incubated for 1 h with primary antibodies (Table S1). All primary antibodies were diluted in PBS containing 2% BSA and 0.01% Tween. Sections incubated with the primary antibodies CD8 or CD31 were further incubated for 1 h with biotinylated goat anti-rat and goat anti-rabbit (Jackson Immunoresearch Laboratories, Seagrove, PA, USA), respectively. Following PBS wash, slides were incubated at room temperature for 30 min with fluorescently-labeled secondary antibody (Abcam) or Cy3-conjugated streptavidin (Jackson Immunoresearch Laboratories). Sections were counterstained with 1 µg/uL of 4′,6-diamidino-2-phenylindole (DAPI) (ThermoFisher Scientific). ScanScope CS (20X air objective, Aperio Technologies) was used for image acquisition while processing and analysis were performed using ImageJ software (NIH). For quantitative CD4^+^ and CD8^+^ analysis, cells within the margins or center areas of pFUS treated-tumors were counted under blinded conditions and compared to TMC tumors. Mean fluorescence intensity was used to quantify immunohistochemical staining for Ki-67 or cleaved-caspase-3. CD31^+^ areas within the tumors were analyzed using a line detection script in MATLAB (Mathworks) [28].

### 2.7. RNA-Seq

RNA was extracted from frozen tumors using PureLink RNA mini kits (Life Technologies, Carlsbad, CA, USA). RNA concentration and purity were determined by Nanodrop ND-1000 (ThermoFisher Scientific) and Experion^TM^ system (Bio-Rad) using Experion RNA StdSens chips (Bio-Rad). Purified RNA was submitted to the National Institutes of Health Intramural Sequencing Center (NISC) and paired-end mRNA sequencing was performed using NovaSeq 6000 (Illumina, San Diego, CA, USA). Sequences were processed in Partek^®^Flow^®^ (Partek Genomics Suite) using the STAR alignment tool (v2.5.3a). Both genes and transcripts were quantified using the Partek E/M quantification tool (Partek Genomics Suite). Transcripts were normalized and filtered to remove transcripts < 30 counts. Statistical comparisons used gene-specific analysis (GSA), comparing each time point to sham-treated tumors (0 h). Raw transcript counts were also analyzed in Gene Set Enrichment Analysis (GSEA) using Molecular Signature Database v7.2 (Molecular Signatures Database), Hallmark Gene sets and Gene Ontology Biological Processes sets (Gene Ontology). Familywise error rate (FWER) *p*-values < 0.05 were used to determine significant enrichment. Cytoscape (v3.8.2) using the Enrichment Map plugin visualized similarity networks.

### 2.8. Statistical Analyses

Data presentation and statistical analyses were performed using GraphPad Prism 8 (GraphPad Prism). Groups requiring multiple comparisons were subjected to one- or two-way repeated-measures analysis of variance (ANOVA) with Bonferroni post-hoc tests. Unpaired non-parametric *t*-tests were used for pair-wise comparisons. Statistical significance was indicated by a *p*-value < 0.05 and the number of animals is indicated for each experiment. Data sample excluding outliers is presented as dot plots, with the average and the appropriate error bars indicated; or as box and whisker plots, wherein boxes denote the upper/lower quartiles with midline displaying the mean values, and whiskers indicate standard deviations (SD) values.

## 3. Results

### 3.1. pFUS Effect on Tumors

Murine B16 and 4T1 flank tumors ~5 mm in diameter were given a single pFUS treatment (Figure 1A) (1.15 MHz at 6 MPa) and tumor volumes were measured daily. Both tumor types treated with pFUS (*n* = 18 tumors) demonstrated decreased growth rates over 5 days compared un-sonicated TMC (*n* = 18 tumors) (Figure 1B,C).

### 3.2. Proteomic Analyses in B16 and 4T1 Flank Tumors Following pFUS

**Figure 2:**
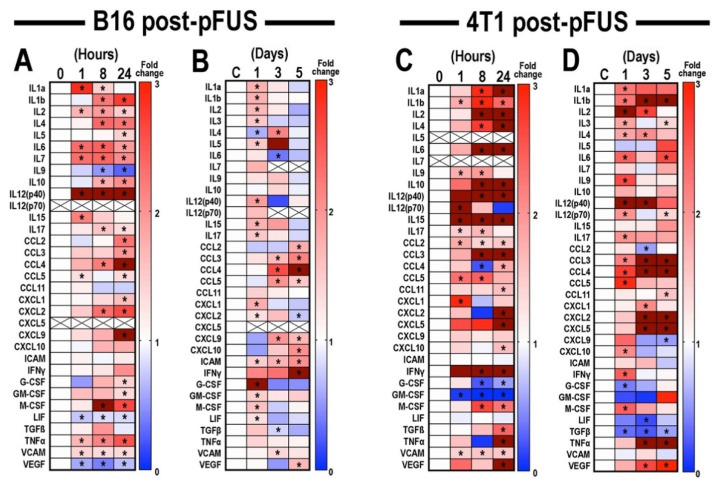
Time-course analysis of proteomic changes in TIME of mouse B16 melanoma and 4T1 breast tumor models (*n* = 6 mice/tumor type/time point) following pFUS. Heat maps depicting fold changes in CCTFs over time of mouse (**A**) B16 melanoma or (**C**) 4T1 breast tumor models 1, 8, or 24 h post-pFUS treatment. For each protein, time points were normalized to values detected on un-sonicated tumors at day 0. See also Figures S1 and S3 for raw data. Temporal proteomic profile of (**B**) B16 and (**D**) 4T1 flank tumor models harvested at 1, 3, or 5 days post-pFUS. Data presented as fold changes between the mean value detected from the pFUS-treated group to their time-matched untreated controls. Increased fold changes are indicated in red, while decreased fold changes are shown in blue. Fold change > 3.1 is displayed as dark red. Time points with *p*-values < 0.05 were considered statistically significant (ANOVA) and marked by asterisks IL: Interleukin; CCL: Chemokine (C-C motif) ligand; CXCL: Chemokine (C-X-C motif) ligand; ICAM: Intercellular adhesion molecule; IFNγ: Interferon-gamma; G-CSF: Granulocyte-colony stimulating factor; GM-CSF: Granulocyte macrophage-CSF; M-CSF: macrophage-CSF; LIF: Leukemic inducible factor; TGFβ: Transforming growth factor-beta; TNFα: Tumor necrosis factor-alpha; VCAM: vascular cell adhesion molecule; and VEGF: Vascular endothelial growth factor. See also Figures S2 and S4 for raw data.

B16 and 4T1 tumors were harvested at 1, 8, or 24 h following pFUS (*n* = 12 tumors/time point) and CCTF and CAM at each time point were measured and compared to sham controls (0 h, *n* = 12 for both tumors). Heat maps of CCTF and CAM expression revealed similarities and differences between pFUS-treated B16 (Figure 2A) and 4T1 tumors compared to sham controls (Figure 2C) (Figures S1 and S3 for raw data). Within the first 24 h post-sonication, there was ~75% (25 of 33) concordance for CCTF and CAM between the B16 and 4T1 tumors with significantly (*p* < 0.05, ANOVA) increased expression profiles consistent with a shift towards anti-tumor (‘hot’) TIME. In both tumor types pFUS increased IL1α, IL1ß, IL2, IL6, IL12p40, IL15, IL17, TNFα, and VCAM. However, there were also significant increases in IL4, IL10, macrophage colony stimulating factor (M-CSF), CCL3, CCL4, CXCL2, and CCL11. Moreover, IL9, leukemia inducible factor (LIF), IFNγ, CCL11, CCL2, CXCL10, VEGF, and TGFβ were unchanged or significantly decreased in B16 tumors. Decreased expression of granulocyte-CSF (G-CSF) and granulocyte macrophage-CSF (GM-CSF) was observed in 4T1 tumors. Other differences include undetected CXCL5 and IL12p70 in the B16 that were present in 4T1 tumors or undetected IL5 and IL7 in the 4T1 that were upregulated in the B16 tumors.

To determine the long-term modulation of TIME by pFUS (Figure 1A), B16 or 4T1 tumor-bearing mice (*n* = 36 pFUS cohort, *n* = 36 TMC) were sonicated on day 0 and harvested on days 1, 3, and 5 days following pFUS (Figure 2B,D, Figures S2 and S4) and compared to un-sonicated TMC. For both B16 and 4T1 tumors, analysis of CCTF and CAM on day 1 post-pFUS recapitulated observations in the initial 24 h time-point (Figure 2A,C), indicating a shift in the CCTF profile towards an inflammatory TIME. By day 3 post-pFUS, the CCTF and CAM in both tumors showed significant increased expression of pro-inflammatory factors including IL1α, IL2, IL12p40, TNFα, and CXCL9 compared to TMC. By day 5, B16 tumors showed significant elevations (*p* < 0.05 ANOVA) for IFNγ, CXCL9, CXCL10 along with CCL2, CCL3, CCL4, CCL5, CXCL2, and VEGF, suggesting a shift toward a less inflamed TIME. In comparison, 4T1 tumors had elevated IL1b, IL3, IL6, IL12p70, CXCL5, CCL11, and TNFα compared to TMC. The variation in expression of CCTF and CAM following pFUS on days 3 and 5 may reflect the heterogeneity of the naïve control tumors during natural progression (Figures S3 and S5) rather than the pFUS effect.

### 3.3. Flow Cytometric Analyses of Tumors, Inguinal Lymph Nodes, and Spleens Post-pFUS

**Figure 3:**
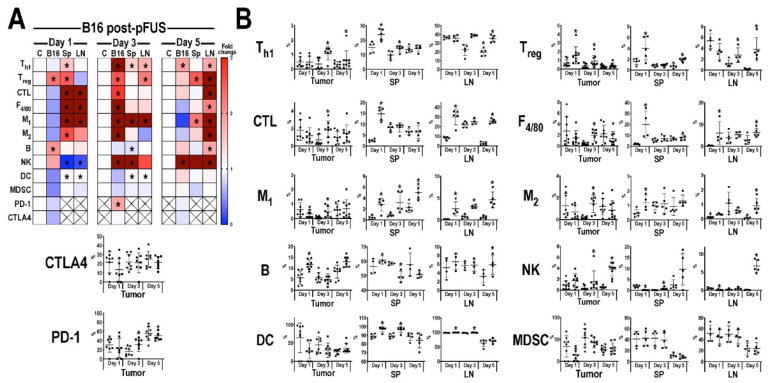
Immune cell profiling following pFUS in B16 tumors. (**A**) Heat map of flow cytometry analysis (*n* = 6 mice/time point) for B16 tumor (B16), spleen (Sp) or lymph node (LN) compared to time-matched control tissues. Tissues were harvested 1 (left), 3 (middle), or 5 (right) days following pFUS; Increased fold changes are indicated in red while decreased fold changes are shown in blue. Fold change > 3.1 is displayed as dark red; (**B**) Quantitative values of immune cell profiles (mean ± SD) of mouse B16 tumor, Sp, or LN, dissected 1, 3, or 5 days following pFUS. *y*-axis represents the relationship between each cell population to the total cells detected; *x*-axis represents days post-pFUS. Asterisks indicate statistical significance (*p* < 0.05; ANOVA) between total cells detected in pFUS-treated mice (black squares) samples to time-matched controls (black circles). Helper T cells (T_h1_), cytotoxic T lymphocytes (CTL), regulatory T cells (T_reg_), natural killer (NK) cells, dendritic cells (DC), F_4/80_ macrophages (M_1_ and M_2_), myeloid-derived suppressor cells (MDSC), cytotoxic T-lymphocyte antigen 4 (CTLA4), programmed death-1 (PD-1) and programmed death-ligand 1 (PD-L1).

**Figure 4:**
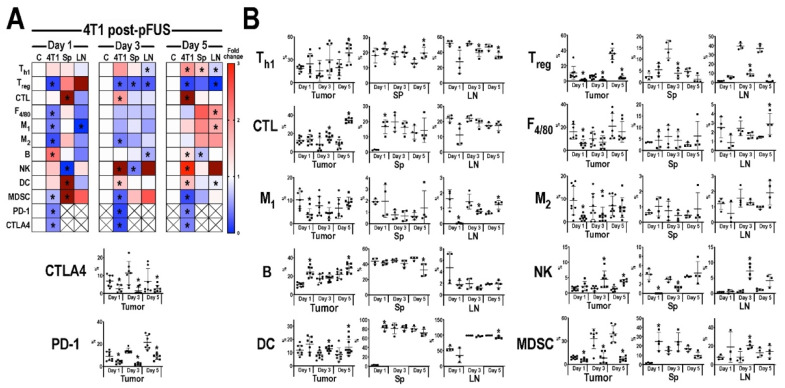
Immune cell profiling following pFUS in 4T1 tumors. (**A**) Heat map of flow cytometry analysis (*n* = 6 mice/time point) results for 4T1 tumor (4T1), spleen (Sp) or lymph node (LN) compared to time-matched control tissues. Tissues were harvested 1 (left), 3 (middle) or 5 (right) days following pFUS; Increased fold changes are indicated in red while decreased fold changes are shown in blue. Fold change > 3.1 is displayed as dark red; (**B**) Quantitative values of immune cell profiles (mean ± SD) of mouse 4T1, Sp, or LN, right, dissected 1, 3 or 5 days following pFUS. *y*-axis represents the relationship between each cell population to the total cells detected; *x*-axis represents days post-pFUS. Asterisks indicate statistical significance (*p* < 0.05; ANOVA) between total cells detected in pFUS-treated mice (black squares) samples to time-matched controls (black circles). Helper T cells (T_h1_), cytotoxic T lymphocytes (CTL), regulatory T cells (T_reg_), natural killer (NK) cells, dendritic cells (DC), F_4/80_ macrophages (M_1_ and M_2_), myeloid-derived suppressor cells (MDSC), cytotoxic T-lymphocyte antigen 4 (CTLA4), programmed death-1 (PD-1), and programmed death-ligand 1 (PD-L1).

The temporal immune cell responses in B16 (Figure 3) and 4T1 (Figure 4) tumors, Sp, and LN at 1, 3, or 5 days post-pFUS were evaluated in tumor bearing-mice from the same cohort study (Figure 1A) by flow cytometry. Relative changes in each immune cell population were calculated as a ratio between pFUS-treated tissues and untreated TMC. On day 1 post-pFUS in B16-bearing mice, the immune cell changes in Sp showed significant elevations (*p* < 0.05 by *t*-test) in CD3^+^CD4^+^ (T_h1_), CD3^+^CD4^+^CD25^+^ (T_reg_), CD3^+^CD4^−^CD8^+^ (CTL), F_4/80+_ (total macrophage population), CD86^+^ (M_1_ macrophages), CD206^+^ (M_2_ macrophages), CD11b^+^ (DC), B cells and decrease in CD3^−^CD335^+^ (NK) cells. The regional LN had similar significant changes in CTL, M1, DC, and NK cells, whereas the B16 tumor was essentially unchanged compared to TMC. By day 3, there was a significant increase of immune cell populations in the B16 tumors (T_h1_, CTL, NK, T_reg_, M_1_, and M_2_ cells) compared to TMC. The Sp and LN had elevated populations of T_h1_, M_1_, NK, and DC along with T_reg_ in the LN. There was also a significant increase in programmed cell death protein 1 (PD-1) but not cytotoxic T-lymphocyte-associated protein 4 (CTLA4) in the tumors. By day 5 post-pFUS, there was continued presence of T_h1_ and NK cells in the tumor. LN also showed significant increases in T_h1_, T_reg_, CTL, NK, B cells, M_1_, and M2 macrophages compared to TMC. There were essentially no changes in the Sp immune cell populations when compared to day 3 post-sonication. In comparison, the response to pFUS in the 4T1 tumors resulted in significant (*p* < 0.05 *t*-test) decrease in immune cell populations (T_reg_, M_1_, M_2_, MDSC) along with PD-1 and CTLA4, except for increases in B cells on day 1 (Figure 4). Sp and LN demonstrated mixed responses, with DC, CTL, and MDSC, increased in the Sp while LN showed decreases in T_h1_, CTL, and M_1_ macrophages compared to TMC. By day 3, the 4T1 tumors had a significant (*p* < 0.05 ANOVA) influx of CTL, NK cells, and DC with decreases in T_reg_, MDSC, PD-1, and CTLA4. There were relatively few differences in the Sp and LN immune cell phenotypes compared to controls except for significant fold changes in NK cell and MDSC in LN for day 3. By day 5 post-pFUS, 4T1 tumors showed increased T_h1_, CTL, B, NK, and DC, with continued decreases in immunosuppressive cells (MDSC, T_reg_) compared to controls. PD-1 and CTLA4 were also significantly decreased. The Sp and LN also had mixed changes compared to the TMC.

In total, by day 5 tumors exhibited a skewing towards an anti-tumor immune cell phenotype comprised of T_h1_ and NK cells in B16 as well as T_h1_, CTL, DC, NK, and B cells in the 4T1 tumors. The shifts in cell populations in Sp and LN demonstrated the most variability on day 5 compared to TMC.

### 3.4. Transcriptomic Analyses of Tumors Following pFUS

**Figure 5:**
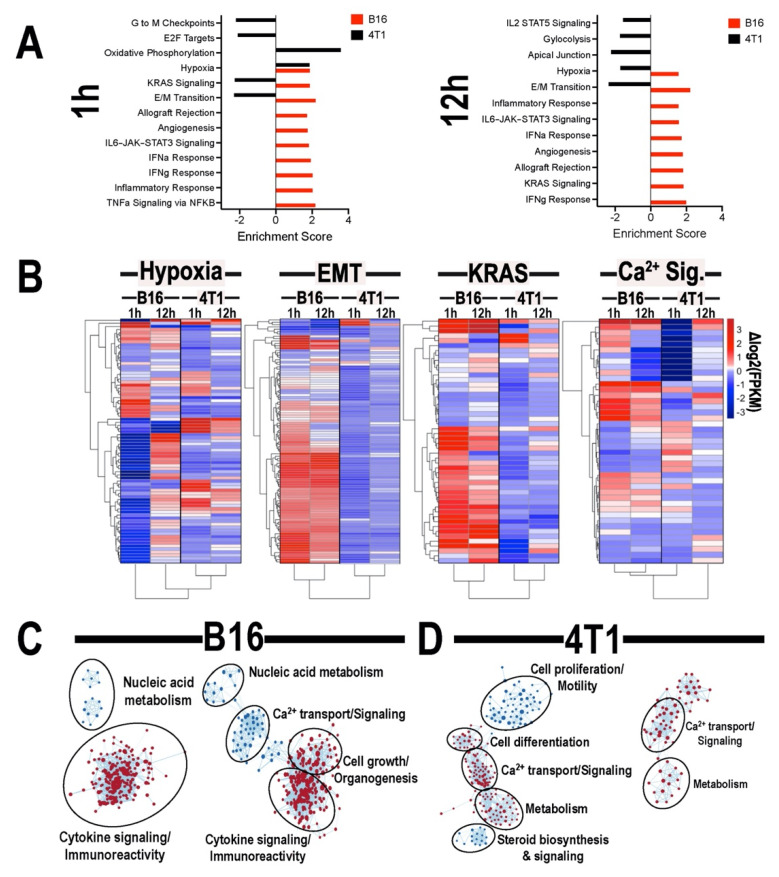
(**A**) Gene ontology (GO) categories of top significantly enrichment obtained from B16 (red bars) and 4T1 (black bars) flank tumors 1 (left) or 12 (right) h following pFUS treatment compared to un-sonicated controls. Log_2_ fold change for each gene is shown in its respective bar. (**B**) Hierarchical clustering of gene expression signature associated with hypoxia, KRAS signaling, epithelial to mesenchymal transition (EMT), and Ca^2+^ signaling. GO of biological processes gene sets for B16 (**C**) or 4T1 (**D**) tumors 1 (left) or 12 (right) h post-pFUS compared to un-sonicated controls. (*n* = 5 mice/tumor type/time point).

RNA-seq was performed on 4T1 and B16 tumors at 1 and 12 h post-pFUS and compared to un-sonicated controls. Normalized transcript counts were evaluated by GSEA for enrichment of Hallmark Gene sets (Figure 5A). At both 1- and 12-h post-sonication, B16 tumors demonstrated positive enrichment of inflammation and cytokine response gene sets, while 4T1 tumors were predominately characterized by negative enrichment of oncogenic, metabolic, and cell-cycle progression gene sets. Kirsten rat sarcoma viral oncogene homolog (KRAS) signaling and epithelial to mesenchymal transition (EMT) were positively enriched in B16 tumors and negatively enriched in 4T1 at both time points. Hierarchical clustering of gene expression changes for genes associated with hypoxia, KRAS signaling, EMT, and Ca^2+^ signaling was performed (Figure 5B) and clustering of treatment groups revealed distinct differences between 4T1 and B16 tumors regarding responses to pFUS. Further GSEA analysis examining enrichment of gene ontology (GO) biological processes gene sets was performed for each tumor type compared to un-sonicated controls and similarity networks were generated (Figure 5C). Several GO gene set for innate immune reactions and cytokine signaling were positively enriched in B16 tumors at both time points and by 12 h post-sonication. GO gene sets corresponding to cell growth and organogenesis are also positively enriched. 4T1 tumors demonstrated positive enrichment for Ca^2+^-dependent signaling processes at both time points. In 4T1 tumors at 1 h post-sonication, gene sets corresponding to cell differentiation were positively enriched, while gene sets corresponding to cell proliferation and steroid hormone signaling were negatively enriched.

### 3.5. Histological Analyses of Tumors Following pFUS

**Figure 6:**
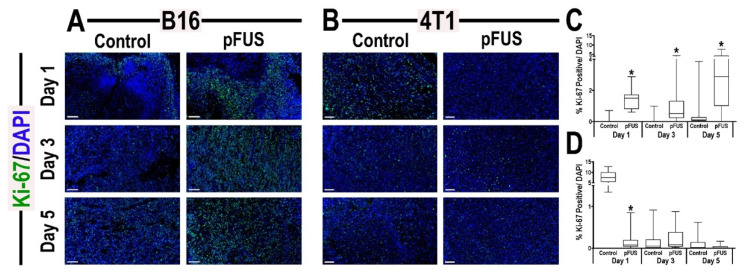
Representative images of Ki-67 localization within sectioned (**A**) B16 melanoma or (**B**) 4T1 breast flank tumor sections 1 (top), 3 (middle), or 5 (bottom) days following pFUS treatment (right) or untreated control tumors (left) (*n* = 6 sections/tumor type/time point). Quantitative analysis of Ki-67 localization within (**C**) B16 tumors or (**D**) 4T1 tumor area. The upper and lower bounds of the boxplots denote the 25th and 75th percentiles, while the midlines indicating the mean values. Whiskers indicate values outside the upper/lower quartile and within standard deviations. Asterisks indicate statistical significance (*p* < 0.05; unpaired *t*-test) between tumor-bearing mice treated with pFUS to time-matched untreated controls. (scale 100 µm).

Based on the gene expression patterns between the tumor types, we investigated cell proliferation and apoptosis using Ki-67 (Figure 6) and cleaved-caspase-3 (Figure S5) immunostaining following pFUS over 5 days. Quantitative analysis of Ki-67 revealed significant increases (*p* < 0.05 by unpaired *t*-test) in B16 at all time points compared to untreated TMC (Figure 6C). However, significant decreases in Ki-67 (*p* < 0.05 by unpaired *t*-test) were detected in 4T1 tumors at 1-day post-pFUS, but no significant differences were observed between 4T1 tumors and TMC on days 3 or 5 days post-pFUS (Figure 6D). pFUS significantly increased (*p* < 0.05 by unpaired *t*-test) activated cleaved-caspase-3 in both the B16 and 4T1 tumors on days 1 and 3 compared to TMC, but no apparent differences were observed at day 5 (Figure S5). The results indicate that pFUS can induce a heterogenous response with proliferation signaling and apoptosis in both tumor types over time. To determine if pFUS exposure results in vascular density changes, B16 and 4T1 tumors were stained with endothelial marker CD31. On days 1 and 3 following sonication, there were significant increases (*p* < 0.05 by unpaired *t*-test) in CD31 expression with no differences on day 5 compared to TMC (Figure S6). Quantifying the numbers of CD4^+^ and CD8^+^ cells within the margin and center of the tumors revealed significant increases (*p* < 0.05 by unpaired *t*-test) in both T_h1_ and CTL on days 3 and 5 in the B16 and only on day 3 for the 4T1 tumors (Figure S7). The differences in observed results between the flow cytometry and immunostaining for CD4^+^ and CD8^+^ cells in both tumors are presumably due to the separate cohorts of mice used for analysis along with the types of fluorescent markers and the numbers of cells counted from a larger volume versus region of interests on 4–6 sections of each tumor type.

## 4. Discussion

Therapeutic strategies for solid malignancies often consist of local interventions, such as surgery or radiotherapy, and/or in combination with systemic approaches (i.e., chemotherapy and immunotherapy) [1,2]. Irrespective of tumor types or applied therapeutic options, the clinical outcomes can be altered by the development of residual resistant cancer cells that can evolve into recurrence or metastases [29,30]. The emergence of a resistant tumor during or after therapy may involve intrinsic genetic drift toward a more malignant undifferentiated phenotype with upregulation of DNA damage repair, checkpoint proteins, T cell exhaustion, and evasion of cell death through the activation of alternative signaling pathways such as Jak/STAT, NFkB, or PI3K/AKT/mTOR pathways [31,32]. Recent investigations clarified that extrinsic factors found in the cellular and molecular composition of TIME are also deeply involved in tumor progression, metastasis, and response to treatment [33,34]. As tumors proliferate, the TIME often shifts to a pro-tumorigenic immunosuppressive I-E phenotype [5]. Therapeutic interventions with the goal of slowing tumor proliferation and metastasis drives TIME towards an inflamed anti-tumor I-I TIME. There is a spectrum of immune-suppressive or anti-tumor molecular and cellular profiles that can be contained within tumors, which most likely contribute to the inability to eradicate tumors or retard growth [4].

The ablative thermal effects of HIFU with or without microbubbles have been shown to release tumor debris, DAMP, and other protein antigens stimulating innate and adaptive immune responses within the tumor [17,35]. Non-ablative pFUS has been shown to improve solid tumor therapy by inducing an anti-tumor immune responses and retarding tumor proliferation, while limiting cellular damage to surrounding normal tissue [36,37,38]. This study revealed that pFUS mechanotransductive effects induced changes in the TIME over 5 days with both similarities and differences between the 4T1 and B16 tumor types. Following pFUS, growth of both tumor types was slowed over 5 days compared to controls. Ki-67 expression was increased by pFUS in B16, but decreased in 4T1. However, activation of pro-apoptotic cleaved-caspase-3 was observed in both tumor types on days 1 and 3, which would support the slowing of growth by day 5 post-pFUS. The pFUS mediated reduction of Ki-67 expression and elevation of cleaved caspase 3 levels have substantial and favorable prognostic implications for patient care from a histopathological perspective. Despite these pathological differences in TIME, the mechanical effects of pFUS in both B16 and 4T1 resulted in increased expression of acute pro-inflammatory CCTF (i.e., IL1, IL2, IL6 IL7, IL15, IL17 TNFα, IFNγ, CCL4, CXCL2, and CXCL10) and CAM (VCAM) as compared to TMC. In addition, the relative decrease in TGFβ in the sonicated tumors compared to TMC would favor an anti-tumor TIME and immune cell response [39]. The changes in CCTF and CAM expression originate presumably from the tumor, stromal, and immune cells that would be consistent with movement towards an I-I TIME. The shift towards a pro-inflammatory TIME with increases in TNFα over the first 3 days following pFUS could also be responsible for the increased expression of CD31 on endothelium reflective of immunological stress within both tumor types [40]. The tumor vascular responses including increased VCAM within 24 h post-pFUS may have also facilitated the immune response. The proteomic analysis, which was limited to primarily an inflammatory panel of CCTF, revealed differences between the two tumor types. Therefore, our results could not be considered encyclopedic as far as reflecting the changes in the TIME following pFUS over time. Furthermore, some CCTFs have dual functions and can either promote or inhibit tumor development and progression depending on intra-tumoral concentrations within the TIME [41,42,43]. The dual functionality of CCTF may partly result in the paradoxical expression detected throughout tumor progression and differences between tumor types. Our results suggest that pFUS may alter the expression of pro-inflammatory CCTF locally at the treated area, that can generate a more robust immunogenic response. However, the discrepancy of immune cell population profiles and proteomic patterns between the B16 or 4T1 tumors emphasizes the profound heterogeneity between the tumors, rather than a function of pFUS effects. Further investigations are needed to optimize the pFUS parameters needed to maximize an immunotherapeutic response in different types of tumors, and between stages of progression in tumors of similar origin that would facilitate a prolonged shift from ‘cold’ towards ‘hot’ TIME.

The transcriptomic responses to pFUS revealed divergent activation of signaling pathways between the two tumor types. Specifically, over 12 h, the EMT and KRAS pathways were positively enriched in the B16 which would be consistent with a more proliferative and malignant phenotype as compared to the control B16 tumors [44,45]. There were also marked differences between the B16 and 4T1 tumors in EMT gene expression at both time points when sonicated tumors were compared to controls. EMT-inducing transcription factors has been associated with more invasive melanoma phenotype, where tumors can shift to a more proliferative and invasive metastatic potential [46,47]. In addition, the changes in KRAS pathway [48] would support a proliferative phenotype within the 12 h following pFUS to the B16 tumors. However, due to the heterogeneity of each tumor type it is difficult to relate genomic changes early on following pFUS to the proteomic and histological changes on days 1, 3, and 5. For the 4T1 tumors, EMT and KRAS pathways were negatively enriched following pFUS compared to controls over the initial 12 h, which would be consistent with slowing tumor growth. Further longer-term follow-up RNA-seq studies will be needed to develop an understanding of how the acute genomic changes affect TIME on day 5 post-sonication. Moreover, single-cell genomic changes within the tumors at early and later time points is warranted to potentially optimize the anti-tumor effects of non-ablative pFUS.

Various cellular and biological immunotherapies have advanced the treatment of human malignancies by augmenting endogenous anti-tumor immune responses [49], thereby shifting the balance from I-E to I-I within the TIME [50,51,52]. Nevertheless, it is increasingly evident that the efficacy of immunomodulatory strategies depends on the presence of a baseline immune response [15]. Thus, many approaches may not be effective, primarily due to the immunosuppressive TIME preventing efficient T cell infiltration and may require additional therapeutic interventions to amplify the immune threshold to result in a more robust therapeutic response [53,54,55,56]. Compared to other therapies, non-ablative pFUS modulates the TIME without thermal damage and results in dynamic changes to immune cell populations in the regional LN and Sp that were different from the targeted tumors [25,26]. By day 5 post-pFUS, there was evidence of both an innate and adaptive immune response with the presence of T_h1_ and NK cells within B16 tumors with the addition of CTL and DC in 4T1 tumors. The observed changes in immune cell populations over 5 days may reflect the ability of pFUS to alter Sp and LN microenvironments through mechanical effects which resulted in tumor debris and/or DAMP release initiating an inflammatory response [26]. The differences in immune cell patterns among the LN, Sp, and tumors over 5 days reinforce the complexity of the underlying mechanisms that influenced tumor growth. The inflammatory response within the TIME was probably responsible for the ultimate priming and increased effector immune cell proliferation and/or transmigration from secondary lymphoid organs. Other ultrasound techniques including thermal ablation, hyperthermia/thermal stress, mechanical forces, and histotripsy have been shown to induce genomic, vascular, and immune cell infiltration in tumors [18,19,57,58]. In the current study, we demonstrated that a single pFUS treatment slowed tumor growth and initiated an innate and adaptive response within the TIME. Further studies should investigate more robust cytotoxic immune cell infiltration and higher immune score [15,59] in the tumors by time with repeated courses of pFUS, with or without chemotherapy or immunotherapy [17]. Previous reports have shown that multiple consecutive days of pFUS coupled with infusion of stem cells resulted in increased numbers in targeted regions [60]. It is plausible that multiple non-ablative pFUS exposures would alter the TIME and cause persistent slowing of tumor growth or unmask tumor-specific antigens that could be targeted by the immune system.

There are several limitations in this study that need to be addressed. The physical effects of the sonication parameters used here require further investigation to identify physical ultrasound mechanisms (acoustic radiation forces (ARF) or cavitation forces) that generate the observed molecular and cellular biological effects in tumors. The parameters used in this study were identified from a previous study that evaluated molecular biological effects in these tumor types over a range of sonication intensities [25]. These parameters represented the lowest ultrasound intensity where pro-inflammatory, anti-tumor molecular and cellular responses were observed. Additionally, we recently reported that these sonication parameters generated endogenous microbubble formation and cavitation in skeletal muscle [61]. We further demonstrated that inflammatory molecular responses in skeletal muscle did not specifically require cavitation, but that such responses were more sensitive to cavitation than ARF. Therefore, further examination of ultrasound physics in these tumor models could help elucidate which forces are biologically transduced into desirable molecular and cellular effects. The proteomic and transcriptomic results were based on bulk analysis of the tumor at specific time-points, consequently limiting the ability to identify which cell population contributes to the CCTF changes following sonication. Specifically, future studies should investigate various live, immune cell populations by high parametric, multicolor immunophenotyping enabled sorting, and perform single-cell RNA sequencing (scRNA-seq) that would provide clues into which specific cell populations that contribute to the anti-tumor effect, and how the ARF alter the cellular metabolic changes translating into an increased expression of CCTF and CAM [3,62]. scRNA-seq data for longer time points out to 5 days post-pFUS may elucidate changes in tumor, stromal, and immune cell populations along with tumor growth. Further histological investigation would be needed to identify the cell population(s) that are Ki-67 positive within the B16 tumors and which cells are undergoing apoptosis in both tumors.

In this study, the immunomodulatory effects of pFUS by flow cytometry were performed on fixed cells extracted from the two tumor types, LN and Sp and resulted in several hundred of samples being processed at days 1, 3, and 5. Unfortunately, due to time constraints live/dead cell determination of samples used for flow cytometry could not be performed. Additional evaluation is warranted to determine the relationship between the shifting of immune cell populations and the molecular alterations within the TIME on days 1, 3, and 5 post-pFUS. Isolation of microvesicles/exosomes and microRNA over time may also provide an added dimension into the transient alterations in the TIME that have been observed with sonication [63,64]. Further evaluation in to release of microvesicles from the tumors [65] may extend our understanding on how non-ablative pFUS may affect tumor growth.

## 5. Conclusions

This study attempts at exploring the complex and dynamic relationship between the immune system and primary tumors and to robustly monitor changes in the immune response. Despite the inherent tumor heterogeneity, we provide a conceptual overview correlating temporal molecular responses to the non-ablative pFUS. The pFUS-induced immunomodulation provided additional novel insights into the mechanisms associated with its anti-tumor immune effect.

## Data Availability

The data that support the findings of this study are available from the corresponding author upon reasonable request.

## Supplementary Materials

The following are available online at https://www.mdpi.com/article/10.3390/cancers13071546/s1, Figure S1: Quantitative values of CCTFs in melanoma B16 tumor flank model 1, 8, or 24 h post-pFUS, Figure S2: Quantitative values of CCTFs in melanoma B16 tumor flank model 1, 3, or 5 days post-pFUS, Figure S3: Quantitative values of CCTFs in breast 4T1 tumor flank model 1, 8, or 24 h post-pFUS, Figure S4: Quantitative values of CCTFs in breast 4T1 tumor flank model 1, 3, or 5 days post-pFUS, Figure S5: Representative merged mosaics of cleaved-caspase 3 localization within tumor sections 1, 3, or 5 days following pFUS treatment, Figure S6: Representative merged mosaics of CD31 localization within tumor sections of 1, 3, or 5 days following pFUS treatment, Figure S7: Representative imaging of CD4 and CD8 localization within tumors’ margin or center area following pFUS treatment. Table S1: A detailed list of antibodies and isotype controls.
