# Supplementary material for: Pulsed-Focused Ultrasound Slows B16 Melanoma and 4T1 Breast Tumor Growth through Differential Tumor Microenvironmental Changes
Source: Cancers (Basel). 2021 Mar 27;13(7):1546. doi: 10.3390/cancers13071546 (PMC8036693; doi:10.3390/cancers13071546)
Supplement: Supplementary file 1 [file cancers-13-01546-s001.pdf]

Supplementary Materials: Pulsed-Focused Ultrasound Slows B16 Melanoma and 4T1 Breast Tumor Growth through Differential Tumor Microenvironmental Changes

Gadi Cohen, Parwathy Chandran, Rebecca M. Lorsung, Omer Aydin, Lauren E. Tomlinson, Robert B. Rosenblatt, Scott R. Burks and Joseph A. Frank

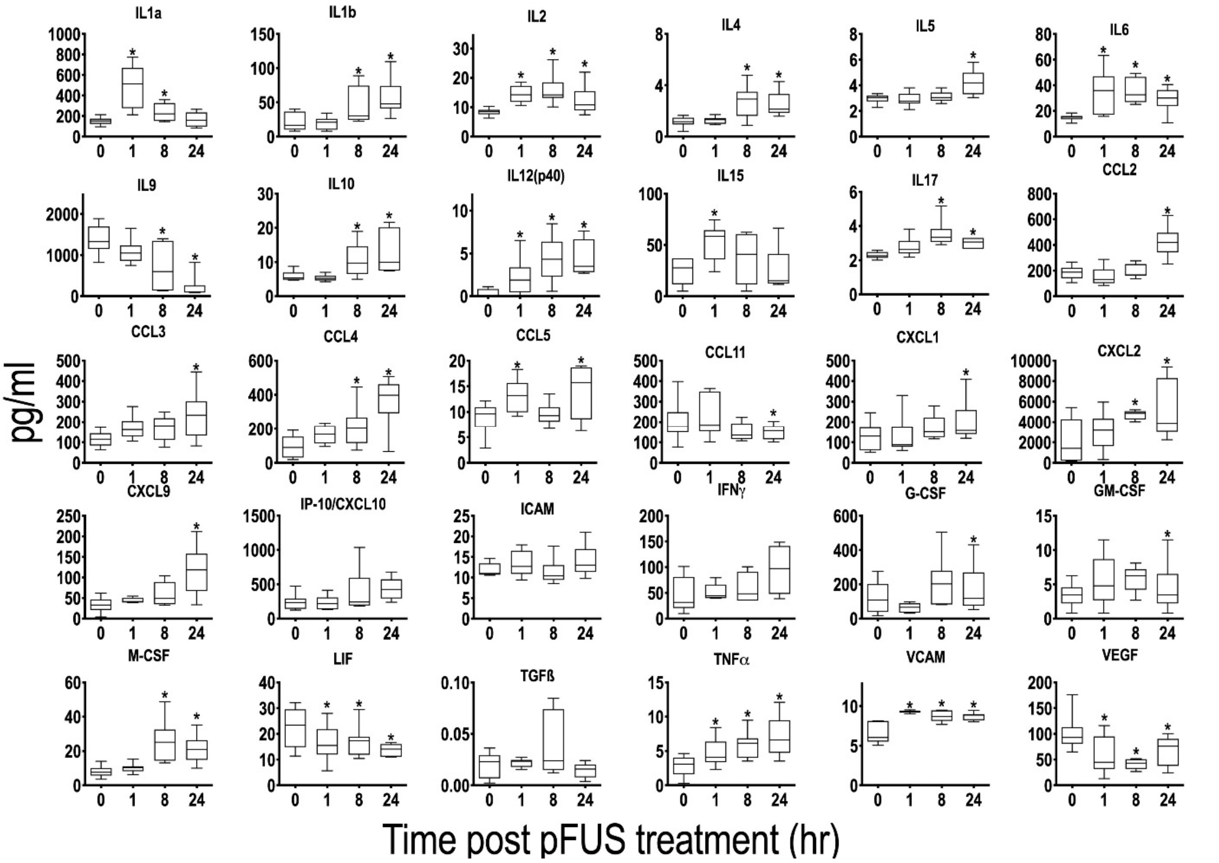

Figure S1. Quantitative values of CCTFs in melanoma B16 tumor flank model 1, 8 or 24 h post-pFUS (n = 6 mice/time point); The y axis represents picograms (pg) per milliliter (ml); the x-axis represents days after reaching ~5mm size in diameter. The upper and lower bounds of the boxplots denote the 25th and 75th percentiles, while the midlines indicate the mean values. Whiskers indicate values outside the upper/lower quartile and within standard deviations. Asterisks indicate statistical significance compared to values detected in untreated control (0 h) ($p < 0.05$; ANOVA).

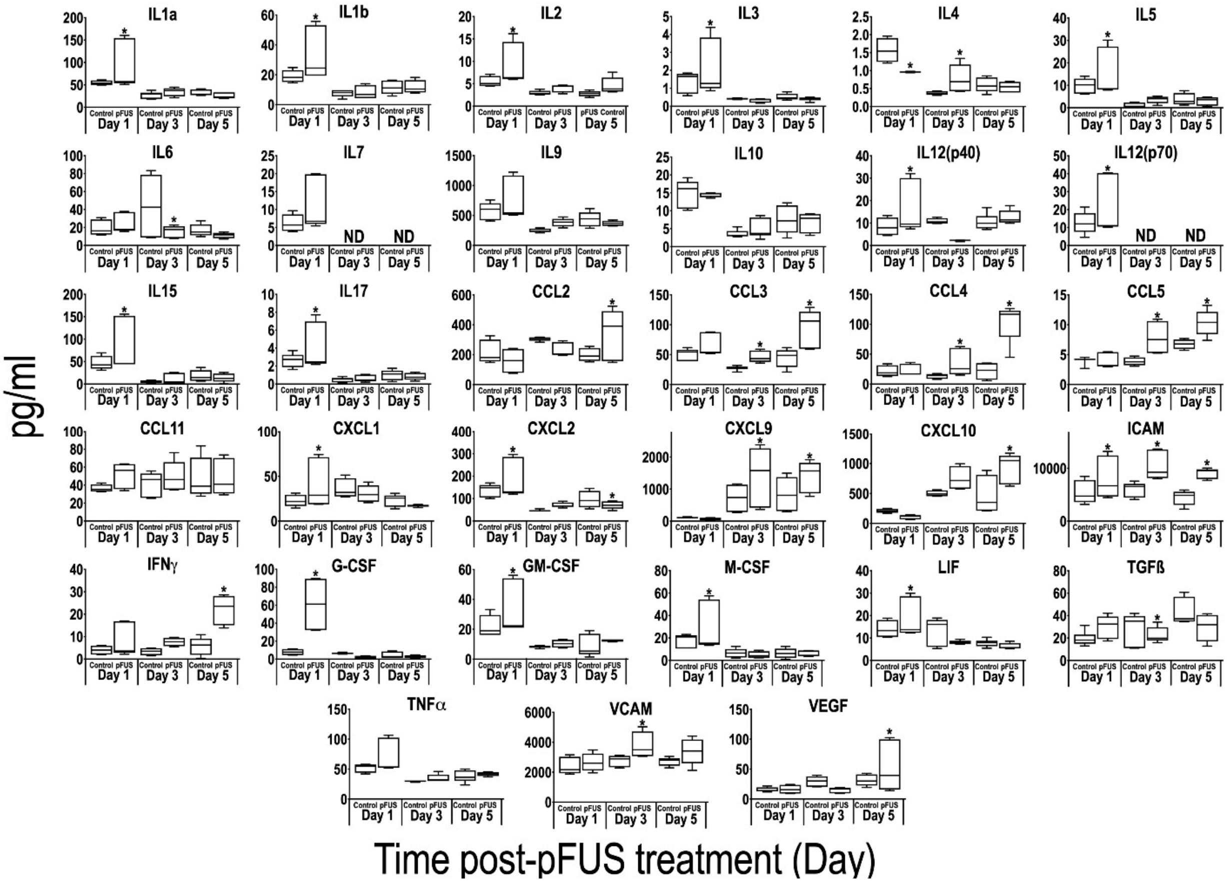

Figure S2. Quantitative values of CCTFs in melanoma B16 tumor flank model 1, 3 or 5 days post-pFUS (n = 6 mice/time point); The y axis represents picograms (pg) per milliliter (ml); the x-axis represents days after reaching ~5mm size in diameter. The upper and lower bounds of the boxplots denote the 25th and 75th percentiles, while the midlines indicate the mean values. Whiskers indicate values outside the upper/lower quartile and within standard deviations. Asterisks indicate statistical significance compared to values detected in the time-matched untreated control ($p < 0.05$; ANOVA).

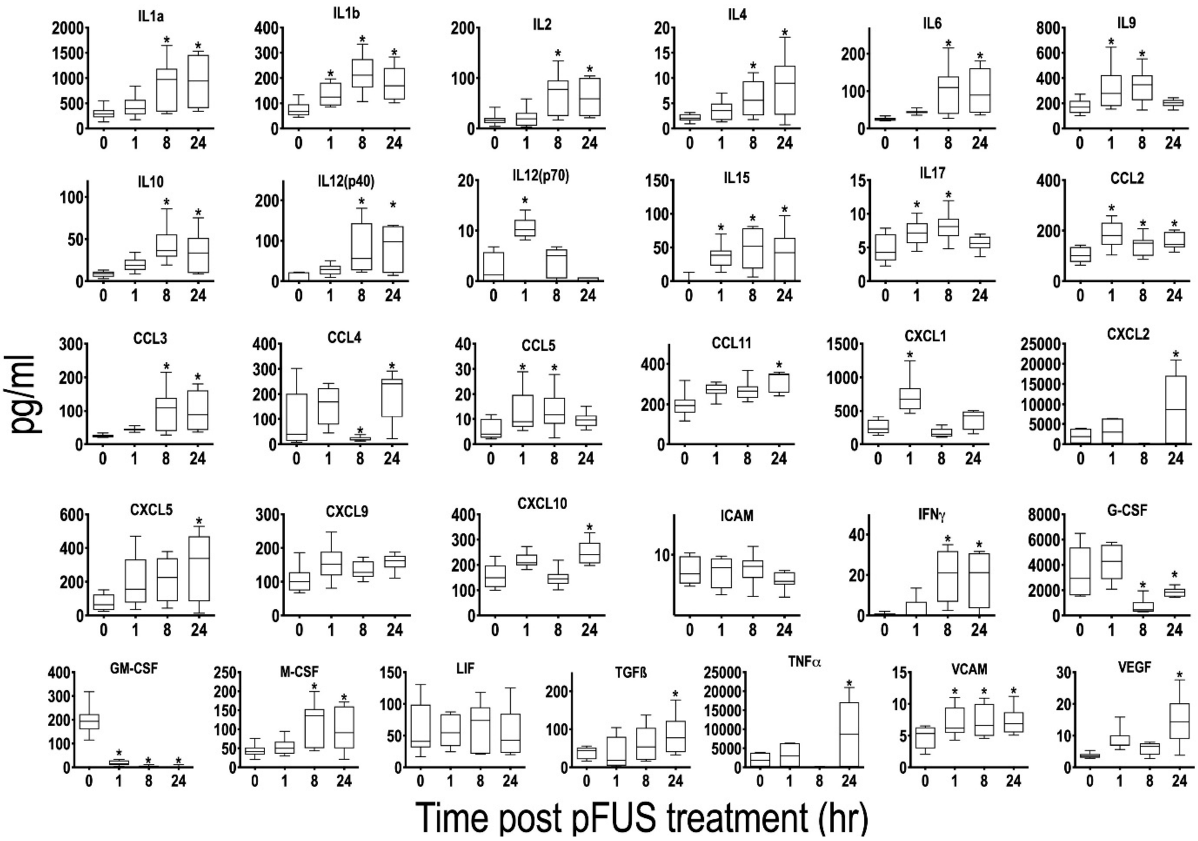

Figure S3. Quantitative values of CCTFs in breast 4T1 tumor flank model 1, 8 or 24 h post-pFUS (n = 6 mice/time point); The y axis represents picograms (pg) per milliliter (mL); the x-axis represents days after reaching ~5mm size in diameter. The upper and lower bounds of the boxplots denote the 25th and 75th percentiles, while the midlines indicate the mean values. Whiskers indicate values outside the upper/lower quartile and within standard deviations. Asterisks indicate statistical significance compared to values detected in untreated control (0 h) ($p < 0.05$; ANOVA).

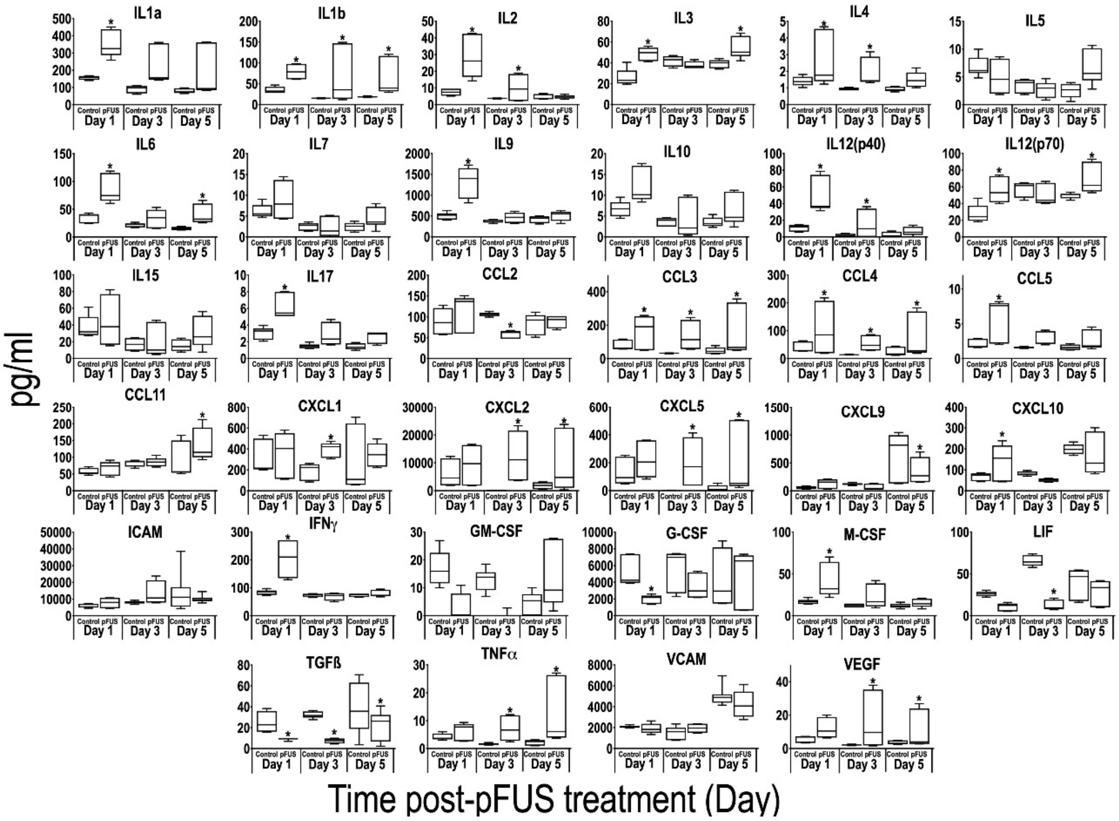

Figure S4. Quantitative values of CCTFs in breast 4T1 tumor flank model 1, 3 or 5 days post-pFUS (n = 6 mice/time point); The y axis represents picograms (pg) per milliliter (mL); the x-axis represents days after reaching ~5mm size in diameter. The upper and lower bounds of the boxplots denote the 25th and 75th percentiles, while the midlines indicate the mean values. Whiskers indicate values outside the upper/lower quartile and within standard deviations. Asterisks indicate statistical significance compared to values detected in the time-matched untreated control ($p < 0.05$; ANOVA).

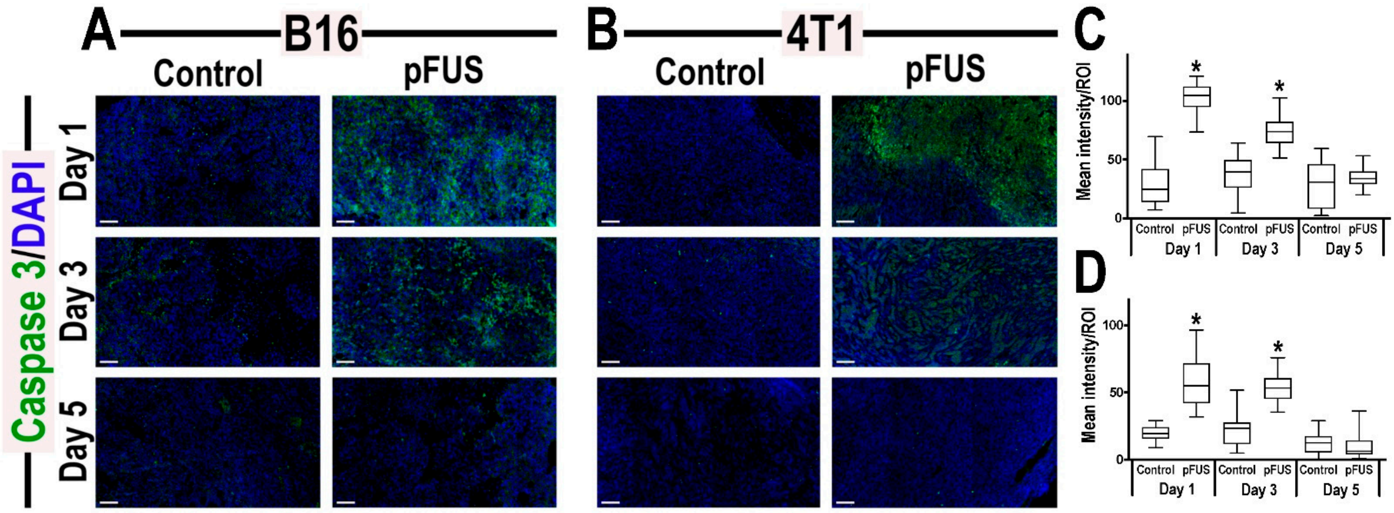

Figure S5. Representative merged mosaics of cleaved-caspase 3 (green) and DAPI (blue) localization within sections of B16 (**A**) or 4T1 (**B**) flank tumors following pFUS treatment (right) or time-matched untreated control (left) (n = 4 sections/tumor type/time point); Quantitative analysis of mean intensity signal detected within a region of interest (ROI) of B16 (**C**) or 4T1 (**D**) tumors; The upper and lower bounds of the boxplots denote the 25th and 75th percentiles, while the midlines indicate the mean values. Whiskers indicate values outside the upper/lower quartile and within standard deviations. Asterisks indicate statistically significant differences ($p < 0.05$; unpaired T-test) between the pFUS treated group to a time-matched untreated control. (scale 100 μm).

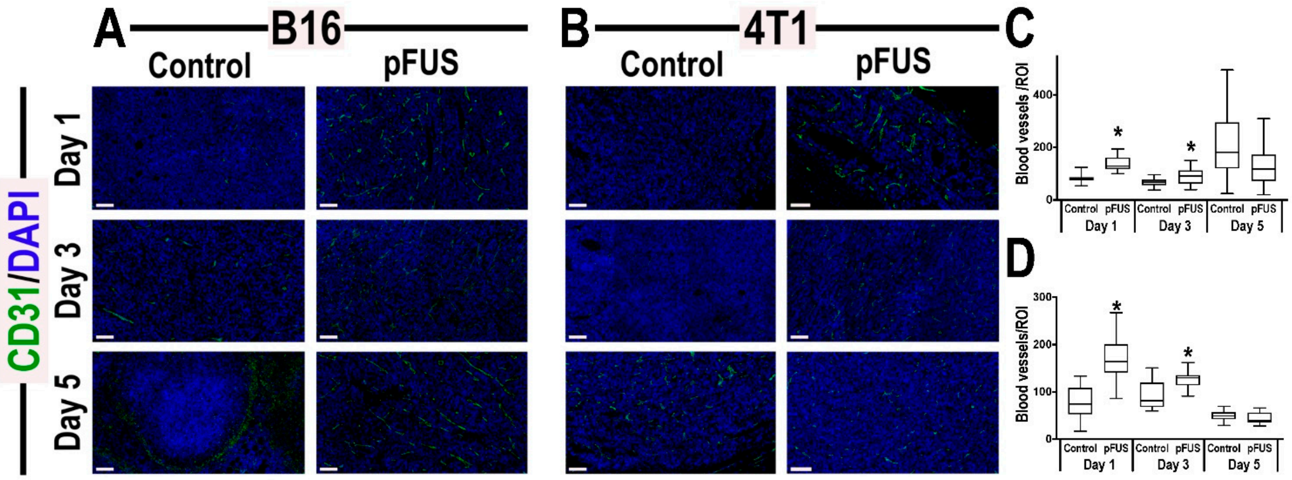

Figure S6. Representative merged mosaics of CD31(green) and DAPI (blue) localization within sections of B16 (**A**) or 4T1 (**B**) flank tumors following pFUS treatment (right) or time-matched untreated control (left) (n = 4 sections/tumor type/time point); Quantitative line detection within a region of interest (ROI) of B16 (**C**) or 4T1 (**D**) tumors; The upper and lower bounds of the boxplots denote the 25th and 75th percentiles, while the midlines indicate the mean values. Whiskers indicate values outside the upper/lower quartile and within standard deviations. Asterisks indicate statistically significant differences (p < 0.05; unpaired T-test) between the pFUS-treated group to a time-matched untreated control. (scale 100 μm).

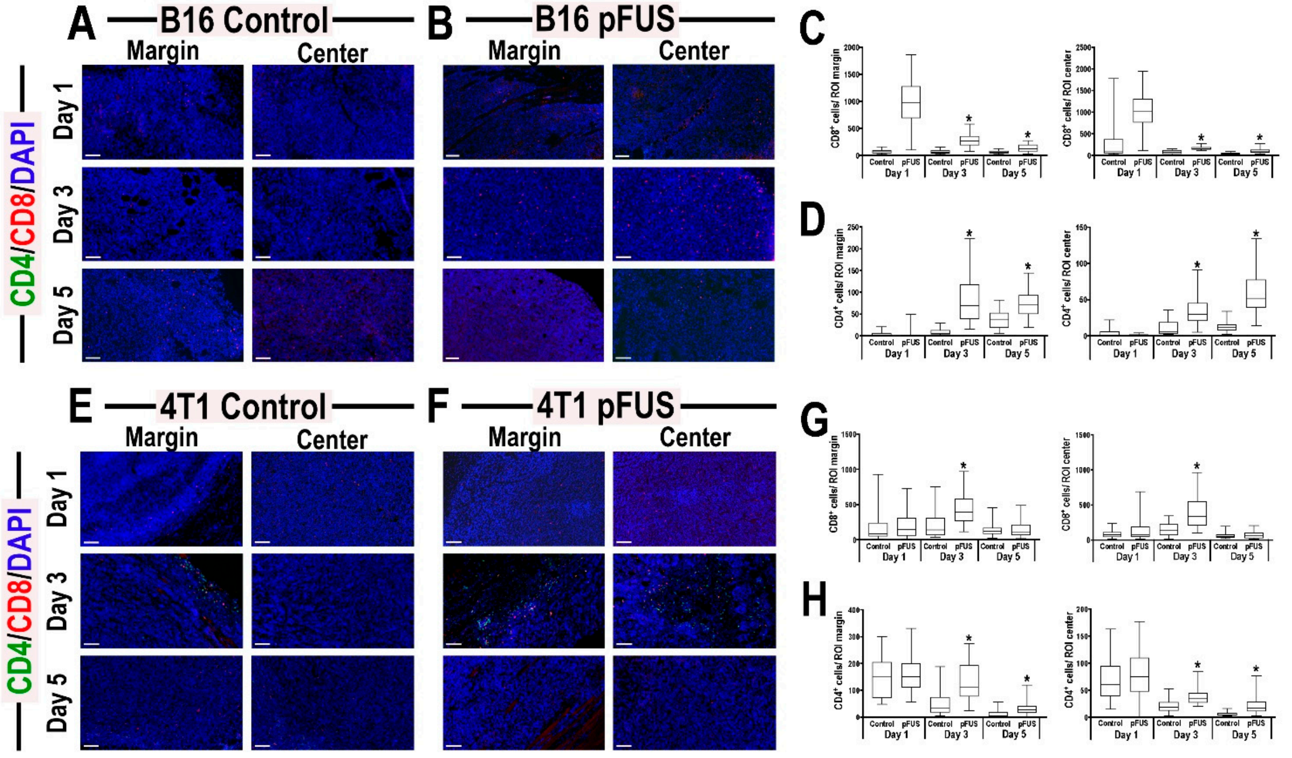

Figure S7. Representative imaging of CD4 (green), CD8 (red) and DAPI (blue) localization of margin (left) or center (right) area within tumors sections following pFUS treatment (n = 6 sections/tumor type/time point); Non-sonicated (**A**) or pFUS treated (**B**) B16 melanoma tumor, 1 (up), 3 (middle) or 5 (down) days post-treatment; Quantitative analysis of time-related changes in $CD8^+$ (**C**) or $CD4^+$ (**D**) cell localization within the center (right) or margin (left) areas of B16 tumors; Non-sonicated (**E**) or pFUS treated (**F**) 4T1 melanoma tumor, 1 (top), 3 (middle) or 5 (bottom) days post-treatment; Quantitative analysis of time-related changes in $CD8^+$ (**G**) or $CD4^+$ (**H**) cell localization within the center (right) or margin (left) areas of 4T1 tumors; The upper and lower bounds of the boxplots denote the 25th and 75th percentiles, while the midlines indicating the mean values. Whiskers indicate values outside the upper/lower quartile and within standard deviations. Asterisks

indicate statistically significant elevations ($p < 0.05$; unpaired T-test) between pFUS treated group to a time-matched untreated control (scale 100 μm).

Table S1. A detailed list of antibodies and isotype controls.

Item	Clone	Cat #	Company
FITC anti-mouse CD3	17A2	100204	BioLegend
FITC anti-mouse F4/80	BM8	123108	BioLegend
FITC anti-mouse CD45	30-F11	103108	BioLegend
PE anti-mouse CD25	PC61	102008	BioLegend
PE anti-mouse CD8a	53-6.7	100708	BioLegend
PE anti-mouse CD206	C068C2	141706	BioLegend
Alexa Fluor 488 anti-mouse/human CD45R/B220	RA3-6B2	103225	BioLegend
PE anti-mouse CD11c	N418	117308	BioLegend
Alexa Fluor® 647 anti-mouse CD335 (NKp46)	29A1.4	137628	BioLegend
APC anti-mouse CD4	RM4-5	100516	BioLegend
APC anti-mouse CD86	GL-1	105012	BioLegend
APC anti-mouse Ly-6G/Ly-6C (Gr-1)	RB6-8C5	108412	BioLegend
PE anti-mouse CD11b	M1/70	101208	BioLegend
APC anti-mouse CD152	UC10-4B9	106310	BioLegend
PE anti-mouse CD274	10F.9G2	124308	BioLegend
Alexa Fluor® 647 anti-mouse CD279 (PD1)	29F.1A12	135230	BioLegend
FITC Rat IgG2b, κ Isotype Control	RTK4530	400606	BioLegend
APC Rat IgG2a, κ Isotype Control	RTK2758	400512	BioLegend
PE Rat IgG1, λ Isotype Control	G0114F7	401906	BioLegend
PE Rat IgG2a, κ Isotype Control	RTK2758	400508	BioLegend
FITC Rat IgG2a, κ Isotype Control	RTK2758	400506	BioLegend
PE Rat IgG2a, κ Isotype Control	RTK2758	400508	BioLegend
APC Rat IgG2a, κ Isotype Control	RTK2758	400512	BioLegend
Alexa Fluor®488 Rat IgG2a, κ Isotype Control	RTK2758	400525	BioLegend
Alexa Fluor®647 Rat IgG2a, κ Isotype Control	RTK2758	400526	BioLegend
FITC Rat IgG2b, κ Isotype Control	RTK4530	400606	BioLegend
PE Rat IgG2b, κ Isotype Control	RTK4530	400608	BioLegend
APC anti-mouse Ly-6G/Ly-6C (Gr-1)	RB6-8C5	108412	BioLegend
PE Armenian Hamster IgG	HTK888	400908	BioLegend
PE Rat IgG2b, κ Isotype Control	RTK4530	400608	BioLegend
Alexa Fluor®647 Rat IgG2a, κ Isotype Control	RTK2758	400526	BioLegend
Rabbit anti-mouse CD4	EPR19514	ab183685	Abcam
Rat anti-mouse CD8	4SM15	14080882	ThermoFisher Scientific
Rabbit anti-mouse Ki-67		ab15580	Abcam
Rabbit anti-mouse CD31		ab28364	Abcam
Rabbit anti-mouse cleaved-caspase 3		ab13847	Abcam
